# Changes upon the gluten-free diet of HLA-DQ2 and TRAFD1 gene expression in peripheral blood of celiac disease patients

**DOI:** 10.1016/j.jtauto.2024.100240

**Published:** 2024-04-09

**Authors:** Mariavittoria Laezza, Laura Pisapia, Benedetta Toro, Vincenzo Mercadante, Antonio Rispo, Carmen Gianfrani, Giovanna Del Pozzo

**Affiliations:** a Institute of Genetics and Biophysics “Adriano Buzzati Traverso”, National Research Council, Naples, Italy; b Gastroenterology, Department of Clinical Medicine and Surgery, University Federico II, Naples, Italy; c Institute of Biochemistry and Cell Biology, National Research Council, Naples, Italy

**Keywords:** Autoimmunity, Inflammation, Celiac disease, Gluten, CD4^+^ T lymphocytes, HLA, TRAFD1

## Abstract

**Background:**

Celiac disease (CD) is a chronic immuno-mediated enteropathy caused by dietary gluten in genetically susceptible individuals carrying HLA (Human Leukocytes Antigen) genes that encode for DQ2.5 and DQ8 molecules. TRAFD1 (TRAF-type zinc finger domain 1) is a gene recently found associated with CD and defined as a master regulator of IFNγ signalling and of MHC class I antigen processing/presentation. There is no specific drug therapy and the only effective treatment is the gluten-free diet (GFD). The great majority of celiac patients when compliant with GFD have a complete remission of symptoms and recovery of gut mucosa architecture and function. Until now, very few studies have investigated molecular differences occurring in CD patients upon the GFD therapy.

**Methods:**

We looked at the expression of both HLA DQ2.5 and TRAFD1 risk genes in adult patients with acute CD at the time of and in treated patients on GFD. Specifically, we measured by qPCR the HLA-DQ2.5 and TRAFD1 mRNAs on peripheral blood mononuclear cells (PBMC) from the two groups of patients.

**Results:**

When we compared the HLA-DQ mRNA expression, we didn't find significant variation between the two groups of patients, thus indicating that GFD patients have the same capability to present gliadin antigens to cognate T cells as patients with active disease. Conversely, TRAFD1 was more expressed in PBMC from treated CD subjects. Notably, TRAFD1 transcripts significantly increased in the patients analyzed longitudinally during the GFD, indicating a role in the downregulation of gluten-induced inflammatory pathways.

**Conclusion:**

Our study demonstrated that HLA-DQ2.5 and TRAFD1 molecules are two important mediators of anti-gluten immune response and inflammatory process.

## Nomenclature

APC: Antigen Presenting cells
B-LCL: B-Lymphoblastoid Cell Line
CD: Celiac Disease
CIITA: Class II Major Hystocompatibility Complex transactivator
EMA: Endomysium antibodies
GFD: Gluten Free Diet
HLA: Human Leukocyte Antigen
MHC: Major Histocompatibility Complex
MyD88: Myeloid differentiation primary response 88
PBMC: Peripheral blood mononuclear cells
LPS: Lipopolisaccaride
IFNγ: Interferon gamma
tTG2: Tissue transglutaminase
TLR: Toll-like receptor
TRAFD1: TRAF-type zinc finger domain 1
TRIF: TIR-domain-containing adapter-inducing interferon-β

## 1 Introduction

Celiac disease (CD) is a chronic enteropathy triggered by an inflammatory reaction induced by gluten ingestion [1,2]. As a consequence of dietary exposure to gluten and activation of gliadin-specific T cells, genetically susceptible individuals develop autoimmune reactions by producing antibodies against tissue transglutaminase (*anti*-tTG2). The CD diagnosis is made in symptomatic HLA-DQ2 or HLA-DQ8 positive subjects with the detection of *anti*-tTG2 confirmed by the presence of *anti*-endomysium antibodies (EMA). The positive serology, the HLA risk factors, and the presence of classic symptoms of celiac disease avoid the requirement of an intestinal biopsy, in children and adults to make a diagnosis of CD [3].

To date, the only effective treatment for CD patients consists of total and life-long elimination of gluten in the daily diet. Following GFD, intestinal morphology and function are restored and patients recover from enteropathy and become negative for *anti*-tTG2 and *anti*-EMA. Several studies have elucidated the key pathogenetic events in CD occurring in the gut mucosa of CD patients eating gluten [4]. The role of the tTG2 enzyme is to convert glutamine residues (a neutral amino acid) to glutamic acid (negatively charged) and the consequence is the high affinity of gluten peptides for HLA-DQ2 or DQ8 molecules, placed on the surface of the APCs, thereby promoting the activation of CD4^+^ T cells. Gluten-specific T lymphocytes mainly produce IFNγ and determine the synthesis of several inflammatory cytokines, that in turn stimulate other cells, including macrophages and fibroblasts, which potentiate the inflammatory response. Thus, T lymphocytes activation induces an inflammatory milieu in the gut mucosa that leads to the destruction of small intestinal architecture, resulting in crypt hyperplasia and villous atrophy. However, mucosal immune responses in CD patients are not limited to the small intestine but involve also peripheral blood. Indeed, T lymphocytes specific for tTG2-deamidated gluten peptides have been found in the blood of untreated CD patients [5], as well as an increased amount of cytokines secreted by Th1 and Th2 lymphocytes have been documented [6,7]. Children with untreated CD show systemic inflammation consisting of an increased production of proinflammatory cytokines in serum, which gets worse after the introduction of gluten in the diet while a decrease of IFNγ was observed during GFD [8,9].

We previously analyzed the expression of CD-associated HLA class II risk alleles in peripheral blood B cells from HLA-DQ2.5 CD children and found a comparable amount of DQ2.5 surface molecules and strength of gluten-specific CD4^+^ T cell response between homozygous and heterozygous subjects [10].

In the current study, we compared the HLA-DQ2 risk gene expression in PBMC (peripheral blood mononuclear cells) from CD patients with active disease and treated patients on GFD, to evaluate the APC capability to present gliadin antigens also during remission.

Moreover, celiac PBMCs were also analyzed for the expression of TRAFD1, a regulator of inflammatory phenotype. TRAFD1 is a transcription factor, which gene has been identified as associated with CD in a recent genome-wide association study from van der Graaf et al. [11]. Through four in silico approaches, the authors found 118 prioritized genes across 50 CD-associated regions and identified TRAFD1 as a *trans*-regulator of 41 genes that were involved in IFNγ signalling and MHC class I antigen processing/presentation pathways. Notably, the TRAFD1 gene encodes FLN29 protein which was indicated as a novel interferon- and LPS-inducible gene, which attenuates TLR-mediated NF-κB signalling by interacting with TRAF6 factor in macrophages. When FLN29 was stably overexpressed in macrophage-like RAW cells, the NF-κB activation and the TNFα production were impaired [12]. FLN29-knockout mice exhibited enhanced induction of inflammatory cytokines and higher susceptibility to LPS- and poly (I: C)-induced endotoxic shock, indicating a TLR-mediated activation of innate immunity [13]. In our study, we analyzed TRAFD1 gene expression in PBMCs from either active or treated patients and correlated its expression with patients HLA genotypes. Furthermore, we measured TRAFD1 transcripts in a monocyte cell line (THP-1) after stimulation with gliadin (the main gluten component) to better investigate its involvement in APC (antigen-presenting cells) function and systemic inflammation occurring in CD.

## 2 Material and methods

### 2.1 Study design and cohort

Patients with active celiac disease, at the time of diagnosis, and patients in follow-up, after at least six months of gluten-free diet, were enrolled at the Department of Gastroenterology of the University Federico II of Naples, from January 1st, 2020 and September 30th, 2023. All subjects enrolled in the study were aged between 25 and 65 years and carried the HLA-DQ2.5 genotype. The inclusion criteria of patients with active disease were the presence of clinical symptoms, antibodies titer *anti*-tTG2> 7 U/mL and *anti*-EMA positive. The inclusion criteria of subjects in follow-up, on GFD from at least 6 months were the absence of clinical symptoms, titer of antibodies *anti*-tTG2 < 7 U/mL and *anti*-EMA negative. Exclusion criteria were: age under 18 years, inability or failure to give informed consent, doubtful or discordant auto-antibody profile, lack of clinical and diagnostic data at diagnosis or at follow-up, and patient's declaration of poor compliance with the gluten-free diet. Peripheral blood withdraws were collected from both groups of patients for the isolation of PBMC from which genomic DNA and total RNA were prepared. The list of 15 patients with active disease and 29 patients on GFD enrolled in the study, with the respective HLA genotype, is reported in Table 1. Three patients affected by the active disease (#CD42, #CD52 and #CD54) were invited to provide another blood withdrawal after six months of GFD to assess the longitudinal changes in TRAFD1 expression during remission.

**Table 1:**
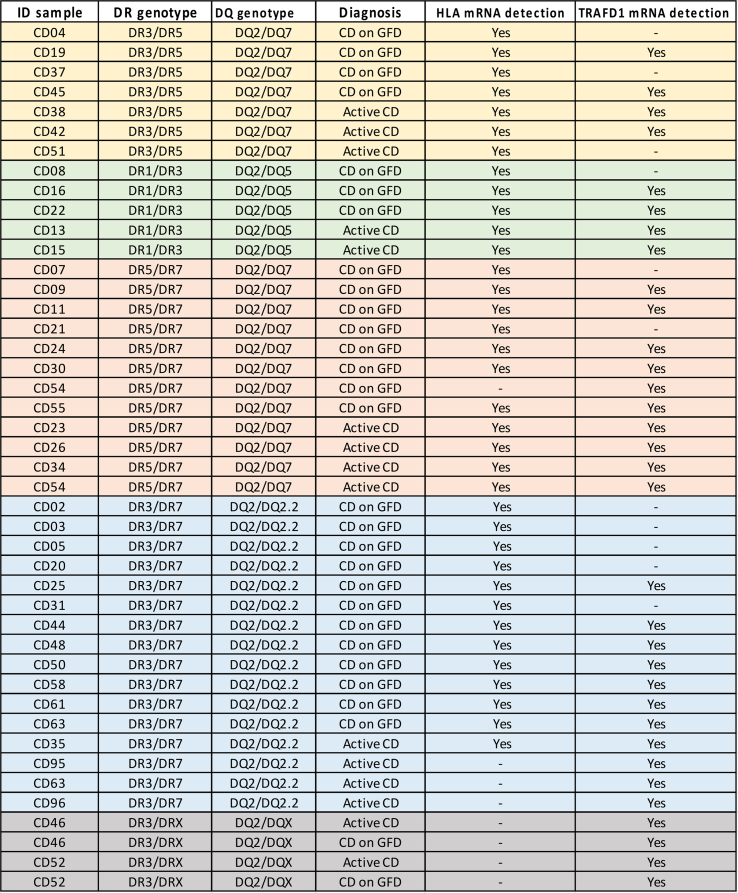
List of patients with their HLA-DR and HLA-DQ genotypes and diagnosis. For each sample, the mRNA value was detected by qPCR.

### 2.2 Ethical approval and consent to participate

The study was approved by the Ethical Committee of the University Federico II of Naples, Italy (protocol n°178/19) and was conducted in accordance with Good Clinical Practice guidelines and the Declaration of Helsinki. The IRB approval date was December 12th, 2019, and the amendment dated December 3rd, 2020, extended the project to December 6th, 2023. A written informed consent for participation in the study was obtained from each patient before the start of the study.

### 2.3 Cells culture

PBMC were isolated from 10 to 15 ml blood samples by Ficoll-Paque PLUS (GE Healthcare BioScience, Milano, Italy) gradient separation. The cells recovered were counted and used either for DNA extraction and gene expression analysis. The cell line THP-1 was cultured in complete RPMI 1640 supplemented with 10 % FBS (Foetal Bovine Serum, Gibco) and 1 % Penicillin/Streptomycin (Lonza). The cells were stimulated with lipopolysaccharide (LPS) from *Escherichia coli* (Sigma) at 5 ng/ml for 3 h and with native gliadin at 50 μg/ml for 16 h. Subsequently, the cells were centrifuged, and the cell pellets were suspended in TRIZOL for RNA preparation or lysis buffer for protein isolation and stored at −80 °C.

### 2.4 Genotyping and gene expression analysis

PBMC (1 × 10^6^) were used to prepare genomic DNA. The HLA genotypes predisposing to celiac disease, were assessed by using a CD-associated gene typing kit (BioDiagene, Palermo, Italy), a commercial diagnostic kit requiring a small amount of blood. All DQ alleles not identified by the BioDiagene kit were determined by the AllSet Gold SSP HLA-DQ Low Res kit (Thermo Fisher Scientific, Monza, Italy). The genotypes of patients are indicated in Table 1.

PBMC (1 × 10^6^) were used to prepare total RNA by TRIZOL reagent. RNA extracted was used for reverse transcriptase reactions, performed by the SuperScript III Reverse Transcriptase enzyme (ThermoFisher), following the manufacturer's protocol. cDNA was used to quantify different transcripts using SYBR Green Master Mix (EuroClone) and the BIORAD Real-Time PCR Detection System instrument. An absolute mRNA quantification was carried out with HLA allele-specific pairs of primers (Supplementary Table 1) and normalized with respect to Glyceraldehyde 3-phosphate dehydrogenase (GAPDH) transcript. Each reaction was conducted in independent triplicates and each experiment was repeated three times. mRNA was prepared by TRIZOL reagent from LPS-stimulated and unstimulated THP-1 cells; TRAFD1 mRNA qPCR was performed by relative quantitation using primers indicated in Supplementary Table 1.

### 2.5 Western blot

Western blot analysis was carried out following total protein cell extraction by RIPA buffer (50 mM Tris-HCl, pH 7.6, 150 mM NaCl, 1 mM MgCl_2_, 0.1 % NP-40). Protein concentration was measured by the Bradford Reagent Spectrophotometer Assay (Bio-Rad Laboratories, Inc.). A total of 20 μg of protein was loaded in 6–10 % acrylamide gel for SDS-PAGE. The gel was blotted on an Immobilon-P PVDF membrane (Millipore) and then treated with *anti*-TRAFD1 (Invitrogen) and *anti*-GAPDH (Sigma) mouse monoclonal antibodies. After incubation with goat anti-mouse secondary antibody, all membranes were developed using an ECL kit (Amersham Bioscience) and exposed to X-ray film.

### 2.6 Statistical analysis

All results are shown as the mean of at least three independent experiments. We confirmed the normal distribution of HLA and TRAFD1 mRNA values, for both active and GFD patients, by Shapiro-Wilck test in R software. Statistical analysis was performed using the unpaired Student's t-test with two-tailed distribution and assuming two samples had equal variance parameters. In the figures, a single asterisk corresponds to p < 0.05 and double asterisks correspond to p < 0.005.

## 3 Results

### 3.1 Expression analysis of HLA-DQ risk alleles

**Fig. 1:**
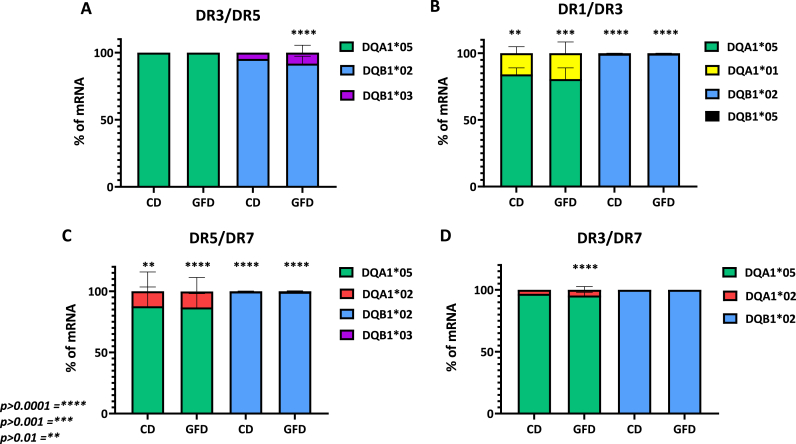
Differential expression of DQA1*05 and DQB1*02 CD risk alleles in PBMC from patients with active CD and patients on GFD. The gene expression of DQA1*05 is shown as a percentage with respect to DQA1*01 (panel B) and DQA1*02 (panels C and D) not associated with the disease. The gene expression of DQB1*02 is shown as a percentage with respect to DQB1*03 (panels A and C) and DQB1*05 (panel B) not associated with disease. Each histogram shows the mean value obtained from different patients stratified according to the genotype, and disease state: DR3/DR5 (n = 4 on GFD, n = 3 with CD, panel A), DR1/DR3 (n = 3 on GFD, n = 2 with CD, panel B) DR5/DR7 (n = 7 on GFD, n = 4 with CD, panel C), DR3/DR7 (n = 12 on GFD, n = 1 with CD, panel D). Statistical analysis was performed using the unpaired Student's t-test with a two-tailed distribution.

The HLA DQ2.5 heterodimers, encoded by CD-associated DQA1*05 or DQB1*02 alleles, present the peptide antigens to gliadin-specific CD4^+^ T lymphocytes. Therefore, the expression of HLA risk genes determines the number of complexes that impact the strength of the autoimmune CD4^+^ T-cell response. DQA1* and DQB1* gene expression was assessed in PBMC, a cell population including antigen-presenting cells and effector T lymphocytes, from both active and GFD patients enrolled in the study. The amount of mRNA transcribed by DQA1* and DQB1* genes, either associated or not to CD, was quantified by absolute qPCR and expressed as a percentage of total transcripts measured. As shown in Fig. 1, a value of 100 % was assigned to the amount of DQA1*05 or DQB1*02 mRNA expressed by DQ2.5 homozygous PBMC, while for DQ2.5 heterozygous patients, we reported the mean percentages of each mRNA within the group with the same genotype. Indeed, the patients were stratified according to their genotype: DR3/DR5 (n = 4 on GFD, n = 3 with active CD,in **panel A),** DR1/DR3 (n = 3 on GFD, n = 2 with active CD, in **panel B),** DR5/DR7 (n = 7 on GFD, n = 4 with active CD, in **panel C**), DR3/DR7 (n = 12 on GFD, n = 1 with active CD, in **panel D)**. The results indicated that the percentage of CD-associated DQA1*05 mRNA varies from 75 % to 95 %, while the amount of mRNA transcribed by DQA1*01 and DQA1*02 alleles, not associated with CD, ranges from 5 % to 25 %, in all heterozygous genotypes. Similarly, the percentage of CD-associated DQB1*02 mRNA ranged from 85 % to 99 % compared to DQB1*03 and DQB1*05 mRNAs, not associated with the disease, that fluctuated from 1 % to 15 %. Our results confirmed the greatest expression of DQA1*05 and DQB1*02 risk alleles in PBMCs in all groups of heterozygous genotypes analyzed, as already demonstrated in B cells [10,14].

**Fig. 2:**
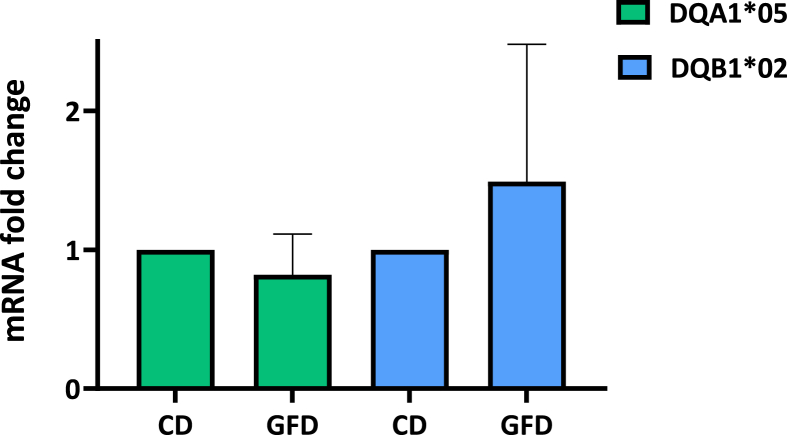
**Comparison of HLA-DQ2 risk alleles expression according to the stage of disease**. The graph shows DQA1*05 and DQB1*02 mRNA fold-change measured in PBMC samples from all patients grouped according to their diagnosis, independently by the specific genotype. No significant differences were observed. Statistical analysis was performed using the unpaired Student's t-test with a two-tailed distribution.

We next grouped the DQA1*05 and DQB1*02 mRNA results obtained in all patients analyzed and plotted them by grouping separately active CD and GFD-treated patients, to compare the HLA risk alleles expression according to the disease state. The results in Fig. 2 demonstrated that there are no significant differences in the expression of the risk alleles between active and treated patients. As the DQA1*05 and DQB1*02 alleles encode for DQ2.5 molecules, that have a key role in presenting antigenic gluten peptides to T cells, these findings provide evidence that PBMCs have the same capability to stimulate adverse T-cell immunity to gluten in celiac patients either on active disease or remission.

### 3.2 Analysis of TRAFD1 expression

**Fig. 3:**
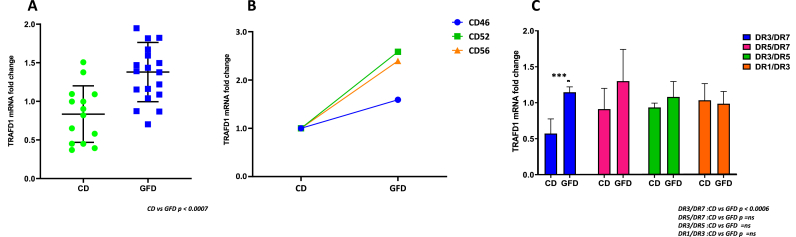
**Analysis of TRAFD1 mRNA expression.** The graph shows TRAFD1 mRNA fold-change measured in PBMC samples from all patients affected by active CD respect patients on GFD (panel A). The increase of TRAFD1 mRNA is shown in three patients after six months of GFD (panel B). In panel C, the value of mRNA is reported in CD and GFD patients stratified according to their genotype. The group with DR3/DR7 genotype shows a significant increment after GFD. Statistical analysis was performed using the unpaired Student's t-test with a two-tailed distribution.

TRAFD1 is a transcription factor, regulator of inflammatory pathways and associated with CD. We investigated if TRAFD1 expression varies during disease follow-up of CD patients. The TRAFD1 mRNA amount, measured by relative qPCR in PBMC, is significantly reduced in the group of patients with active disease with respect to patients on GFD, independently by their genotype (Fig. 3 **panel A**) (p < 0.0007). Then we monitored the TRAFD1 mRNA variation during remission. We prepared PBMC from patients at diagnosis and after six months of GFD for the three of them (patients #CD42, #CD5 and #CD54). As shown in Fig. 3 **panel B**, we observed a 1.5–2.5 fold-increase of TRAFD1 mRNA after GFD when the specific antibodies were negativized and the clinic manifestation recovered, according to the data obtained from the entire cohort analyzed. These results, obtained at different times from the same patient, allowed the monitoring of TRAFD1 mRNA and confirmed its contribution to the resolution of inflammation.

Finally, we analyzed the TRAFD1 expression in relation to the HLA genotype of patients. All groups of DQ2.5 heterozygous patients, with DR5/DR7, DR3/DR5 and DR1/DR3 genotypes, showed different expressions between active CD and GFD patients but only the group carrying DR3/DR7 genotype, associated with the highest risk of pathology, showed a significant increase of TRAFD1 expression after disease remission (Fig. 3 **panel C**).

### 3.3 Effect of gliadin stimulation on TRAFD1

**Fig. 4:**
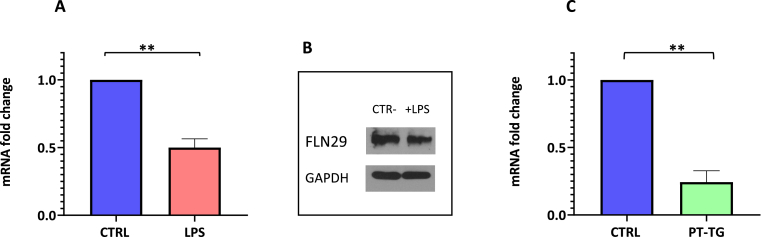
**Variation of TRAFD1 expression in THP-1 cell following LPS or gliadin stimulation**. Panels A and C show TRAFD1 mRNA decrease after LPS and gliadin stimulation, respectively, in the THP-1 cell line. The Western blot of panel B confirmed the downregulation of FLN29 protein after LPS stimulation.

We used the THP-1 cell line to analyze the effect of gliadin stimulation on TRAFD1 expression. Firstly, we demonstrated that LPS stimulus, a classical agonist of inflammation, affects TRAFD1 expression. In fact, in Fig. 4 **panel A**, we observed a decrease in TRAFD1 mRNA amount in LPS-stimulated cells [11,15]. Moreover, we verified that these changes in TRAFD1 expression were associated with the reduction of FLN29 protein synthesis, as indicated in **panel B**. These preliminary experiments suggested that the THP-1 cell line is a good model to verify the inflammatory role of gliadin. Upon gliadin stimulation of THP-1 cells, we observed a significant decrease (p < 0.05) of TRAFD1 mRNA with respect to the unstimulated cells (**panel C**). In conclusion, our results revealed that gliadin stimulation determines an inflammation status that affects the expression of FLN29 proteins consequent to the decrease of TRAFD1 mRNA amount.

## 4 Discussion

Celiac disease is a complex autoimmune enteropathy whose aetiology is determined by a combination of genetic factors (HLA-DQ2 and DQ8 genes), environmental factors (i.e., gluten diet), and cofactors (e.g., microbiota alterations, viral infections, drugs, and winning age). It has been well characterized that gluten proteins, following digestion by tissue transglutaminase 2, stimulate both adaptive and innate immune responses, in the small intestine of CD patients. More specifically, the gluten immunogenic peptides bind with high affinity to the HLA-DQ2 or HLA-DQ8 molecules expressed on the surface of APC and stimulate the activation of antigen-specific CD4^+^ Th1 cells.

In our previous works, we demonstrated the differential expression of CD-associated HLA class II risk alleles, DQA1*05 and DQB1*02, with respect to non-CD-associated ones, in immortalized B cells from DQ2.5 heterozygous children with CD. Moreover, we showed that the expression of the DQ2.5 molecule, encoded by the risk alleles, influences the activation strength of gliadin-specific CD4^+^ T cells [10,14]. In the current work, we compared the expression of the same risk alleles in APC from the blood of both patients with active disease and in remission on GFD, these latters characterized by *anti*-EMA and *anti*-tTG2 negative titers. The PBMC are a heterogenous cell population containing different types of APC, such as monocytes, B lymphocytes and dendritic cells and we verified if circulating APCs from patients on GFD have the same capability to present gliadin antigens compared to APC from patients with active disease. We confirmed the differential expression of mRNAs transcribed by risk alleles with respect to non-CD-associated alleles in the heterozygous patients, as previously demonstrated for B cells. The novelty of the current work is the evidence that the DQA1*05 and DQB1*02 alleles expression did not vary between patients with active disease and patients on GFD. These findings have important implications as a comparable mRNA amount results in a similar density of DQ2.5 molecules on the APC surface, with the consequence that circulating APC from GFD patients have the same capability to present the HLA-DQ2.5-gliadin complexes as APC from patients with active disease. Similar DQ2.5 genes and molecules expression was demonstrated in CD patients despite the IFNγ serum concentration that is reported to be persistently elevated in patients with active disease both in the lamina propria and peripheral blood [8,9], whilst, in patients on GFD, the frequency of circulating IFNγ-releasing cells increase upon gluten challenge [16].

IFNγ promotes the transcription of HLA class II genes in non-professional APC, such as monocytes and macrophages, while dendritic cells and B lymphocytes constitutively express HLA class II molecules through a complex molecular pathway that includes CIITA transactivator [17]. The HLA class II expression in these cell subsets activates a feed-forward loop wherein IFNγ-producing CD4^+^ T cells induce myeloid HLA class II molecules, which in turn amplifies CD4^+^ Tcell responses. For this reason, we investigated if an inflammatory milieu induced by gluten consumption might regulate the expression of HLA-DQ2.5 in PBMC in active patients with respect to patients on GFD [6].

The innate immune system is also involved in the earliest events of CD pathogenesis, through TLRs-downstream activation [18] that, through MyD88 and TRIF-dependent pathways, allows NF-κB to translocate into the cell nucleus and activate transcription of several genes encoding a proinflammatory cascade of cytokines and chemokines. A gene involved in this inflammatory response is TRAFD1, whose overexpression in monocyte/macrophage cells resulted in a suppression of TLR signalling, dampening the inflammatory responses. Otherwise, a depletion of TRAFD1 in human cells or KO mice determines an increased susceptibility to LPS with amplified production of pro-inflammatory cytokines [12,13].

More recently, TRAFD1 has been associated with celiac disease and defined as a novel master regulator, involved in the regulation of a set of genes enriched for two major pathways of immune activation, IFNγ signalling and antigen processing/presentation [11]. The authors of this study suggested for the first time a role of TRAFD1 in signalling response to both type I and type II IFNs that affects the downstream activation of the NF-κB pathway.

In the present work, we observed that TRAFD1/FLN29 expression, in the THP-1 cell line is reduced not only by LPS but also by gliadin stimulation. To deepen the role of TRAFD1 in inflammation, we next measured TRAFD1 mRNA in PBMC from both acute and GFD celiac patients. Interestingly, we found a higher amount in patients in remission with respect to patients with active CD.

It has been reported that the modulation of TRAFD1 represents a clear indication of its role in TLR signalling and NF-κB activation, which in turn controls the transcription of several genes encoding a proinflammatory cascade of cytokines and chemokines [12]. The low expression in active CD is indicative of NF-κB activation while the high expression of TRAFD1 in GFD patients is associated with the NF-κB pathway downregulation, as well as the decrease of the innate and adaptive immune response against gluten [19]. Very attractive is the significant difference in the TRAFD1 expression among CD and GFD patients carrying the DR3/DR7 genotype, which is associated with the highest CD risk, indicating potential involvement of this factor in the genetic profile that contributes to the pathology predisposition.

Much evidence suggests that gliadin-induced immune response may pass through TLRs-dependent pathways that induce the production of some pro-inflammatory cytokines by innate immune cells.

In the lamina propria, the interaction of macrophages with gliadin peptides leads to a pro‐inflammatory cascade that enhances the interaction of T cells with APCs, through MyD88 signalling [19]. MyD88 signalling leads to the activation of NF‐κB in enterocytes, which prompts pro‐inflammatory cytokines transcription contributing to inflammation. Palova‐Jelinkova et al. [20] reported that whole PBMC and purified monocytes from celiac patients responded to pepsin-digested gliadin fraction by a robust secretion of IL-1β, suggesting that gliadin contains not only T-cell epitopes but other peptides that could play a role as adjuvants activating the innate immune system via TLR2/4/MyD88 signalling pathway.

Another study [21] demonstrated that the 33-mer gliadin peptide in murine macrophages induces an overexpression of NF-κB via TLR2/TLR4-dependent pathways supporting the hypothesis that gliadin peptides may directly activate TLRs-mediated mechanisms of innate inflammation. Nanayakkara et al. [22] showed that gliadin peptide p31–43 can induce the IFNα-mediated innate immune response in CaCo-2 cells by activating the TLR7, an endosomal receptor that recognizes viral mRNAs. Upon activation, TLR7 interacts with MyD88, recruits the MAPKs and leads to NF-κB activation. Finally, Junker et al. showed that α-amylase/trypsin inhibitors (ATIs) are strong inducers of innate immune responses in human and murine macrophages, monocytes, and DCs. ATIs are a group of proteins contained in the wheat and other toxic cereals activating the TLR4–MD2–CD14 complex and stimulating the release of pro-inflammatory innate cytokines in isolated cells and intestinal biopsies from mice, as well as from celiac and non-celiac patients [23].

In summary, we demonstrated that high expression of TRAFD1 is associated with GFD, a condition in which the NF-κB pathway is downregulated and the innate and adaptive immune response against gluten is decreased. Otherwise, lower TRAFD1 expression is revealed in PBMC from patients with active diseases in which the NF-κB activation has been extensively demonstrated. In conclusion, TRAFD1/FLN29 may represent a direct biomarker of celiac disease remission and compliance with a gluten-free diet in treated patients.

## 5 Conclusions

We present data regarding the expression of HLA-DQ2.5 and TRAFD1 genes in celiac disease patients on active disease or remission. HLA-DQ2.5 expression does not vary after recovery of symptomatology, indicating that the introduction of gluten antigen during GFD may again stimulate adaptive immune response and activate circulating antigen-specific CD4^+^ T cells. The TRAFD1 gene is an important mediator of the inflammatory process and could be a useful biomarker of celiac disease remission and compliance with a gluten-free regimen in treated patients.

## Funding

This study was supported by 10.13039/501100004462CNR project FOE 2021 DBA.AD005.225 to GDP and CG, by Grant Project 10.13039/501100014566FC (Italian 10.13039/501100014566Celiac Disease Foundation) Investigator N. IG_002_FC_2020 to CG, by triennial Fellowship of Italian Celiac Disease Foundation to ML (Fellowship_ 6_FC_2019).

## Informed consent statement

Informed consent was obtained from all subjects involved in the study.

## CRediT authorship contribution statement

**Mariavittoria Laezza:** Investigation, Formal analysis, Data curation. **Laura Pisapia:** Software, Investigation, Formal analysis. **Benedetta Toro:** Investigation, Data curation. **Vincenzo Mercadante:** Software, Methodology. **Antonio Rispo:** Supervision, Data curation. **Carmen Gianfrani:** Writing – review & editing, Writing – original draft, Funding acquisition. **Giovanna Del Pozzo:** Writing – review & editing, Writing – original draft, Funding acquisition, Conceptualization.

## Declaration of competing interest

The authors declare that they have no known competing financial interests or personal relationships that could have appeared to influence the work reported in this paper.

## Data Availability

The data that has been used is confidential.

## Appendix A Supplementary data

Supplementary Table 1 Sequence of primers used for qPCR Supplementary Table 1
