# Supplementary material for: Changes upon the gluten-free diet of HLA-DQ2 and TRAFD1 gene expression in peripheral blood of celiac disease patients
Source: J Transl Autoimmun. 2024 Apr 9;8:100240. doi: 10.1016/j.jtauto.2024.100240 (PMC11060953; doi:10.1016/j.jtauto.2024.100240)
Supplement: Supplementary Table 1 — Sequence of primers used for qPCR [file mmc1.docx]

| ***Primers used for qRT-PCR*** | | |
| --- | --- | --- |
| ***Gene*** | ***Primers*** | ***Sequences*** |
| **GAPDH** | GAPDH-FW  GAPDH-RW | AACGGATTTGGTCGTATTGGGC  TCGCTCCTGGAAGATGGTGATG |
| **HLA-DQA1*01** | DQA101-FW  DQA1-RW | CGGTGGCCTGAGTTCAGCAA  GGAGACTTGGAAAACACTGTGACC |
| **HLA-DQA1*02** | DQA102-FW  DQA1-RW | AAGTTGCCTCTGTTCCACAGAC  GGAGACTTGGAAAACACTGTGACC |
| **HLA-DQA1*05** | DQA105-FW  DQA1-RW | TGGTGTTTGCCTGTTCTCAGAC  GGAGACTTGGAAAACACTGTGACC |
| **HLA-DQB1*02** | DQB102-FW  DQB1-RW | TCTTGTGAGCAGAAGCATCT  CAGGATCTGGAAGGTCCAGT |
| **HLA-DQB1*03** | DQB103-FW  DQB1-RW | CGGAGTTGGACACGGTGTGC  CAGGATCTGGAAGGTCCAGT |
| **HLA-DQB1*05** | DQB105-FW  DQB1-RW | ACAACTACGAGGTGGCGTACC  CAGGATCTGGAAGGTCCAGT |
| **TRAFD1** | TRAFD1-FW  TRAFD1-RW | GCTGTTAAAGAAGCATGAGGAGAC  TTGCCACATAGTTCCGTCCG |
